# Genotyping of Jujube (*Ziziphus* spp.) Germplasm in New Mexico and Southwestern Texas

**DOI:** 10.3390/plants12132405

**Published:** 2023-06-21

**Authors:** Dikshya Sapkota, Dapeng Zhang, Sunchung Park, Lyndel W. Meinhardt, Shengrui Yao

**Affiliations:** 1 Department of Plant and Environmental Sciences, New Mexico State University, Las Cruces, NM 88003, USA; dikshya@nmsu.edu; 2 Sustainable Perennial Crops Laboratory, USDA-ARS, Beltsville Agricultural Research Center, Beltsville, MD 20705, USA; dapeng.zhang@usda.gov (D.Z.); sunchung.park@usda.gov (S.P.); lyndel.meinhardt@usda.gov (L.W.M.); 3 Sustainable Agriculture Sciences Center, New Mexico State University, Alcalde, NM 87511, USA

**Keywords:** germplasm, single nucleotide polymorphisms, synonymous genotypes, genetic diversity, *Ziziphus jujuba* Mill., *Ziziphus spinosa* Hu.

## Abstract

Since the early 19th century, a substantial amount of jujube (*Ziziphus* spp.) germplasm has been introduced from China and Europe into the United States. However, due to a lack of passport data, cultivar mislabeling is common and the genetic background of the introduced germplasm remains unknown. In the present study, a low-density SNP array was employed to genotype 204 jujube trees sampled from multiple locations in New Mexico, Texas, Missouri, and Kentucky. Multilocus matching of SNP profiles revealed a significant rate of genetic redundancy among these jujube samples. A total of 14 synonymous groups were detected, comprising 48 accessions. Bayesian clustering analysis and neighbor-joining tree partitioned the US jujube germplasm into two major clusters. The first cluster included cultivated genotypes (*Ziziphus jujuba* Mill.), whereas the other major cluster comprised the wild/sour jujube (*Ziziphus spinosa* Hu.). The results also revealed a unique jujube population at Fabens/Tornillo, Texas, and a semi-naturalized population at Tucumcari, NM. These findings will provide valuable guidance to jujube growers and researchers on the effective utilization of jujube germplasm in the horticultural industry.

## 1. Introduction

Jujube (*Ziziphus jujuba* Mill.), also called Chinese date or red date, is native to China [1,2] and belongs to the buckthorn family Rhamnaceae [3,4]. The genus *Ziziphus* comprises approximately 135 plant species [5,6]. Plants of this genus are thorny shrubs or small trees that are mainly found in warm–temperate and subtropical regions in the world [7]. Chinese jujube has been cultivated for at least 4000 years, and archaeological findings indicate it was utilized 7700 years ago in China [8]. Sour jujube (*Z. Spinosa* Hu.) trees in the wild with large fruit and good flavor were domesticated in the past, and consequently, the current jujube cultivar that we know today originated from these ancient selections [9]. Due to its high vitamin C, cyclic AMP, phenolics, flavonoids, natural colorant properties, and mineral content (particularly potassium and iron), as well as biologically active compounds, Chinese jujube is used both as a fruit and herbal medicine for its dry fruit and seeds [10,11,12,13,14,15,16]. Due to its higher tolerance for drought and salinity and lower demand for irrigation and fertilization, jujube is considered an important cash crop for arid and semi-arid regions [3,17]. Along with the large-scale commercial production in China and South Korea, it has been gaining eminence in the US, Australia, and other countries [18].

Jujube seedlings were first imported from Europe to Beaufort, North Carolina, the United States (U.S.) by Robert Chisholm in 1837. In 1876, Rixford brought jujubes from southern France to California’s Sonoma Valley and neighboring states [19]. The United States Department of Agriculture’s Agricultural Explorer, Frank N. Meyer, introduced the first group of commercial cultivars to the Plant Introduction Field Station at Chico, California (CA), in 1908 [20,21]. Later, those cultivars were distributed to other USDA stations in Texas (TX), New Mexico (NM), Oklahoma, Georgia, and Florida [21]. In the 1990s and 2011, more jujube cultivars were directly imported from China and other jujube-growing nations [9]. With frequent international travel, jujube cultivar importation without documents also exists. Jujube cultivars commercially available now include ‘Li’, ‘Lang’, ‘Sugarcane’, ‘Sherwood’, ‘GA866’, ‘Shanxi Li’, and ‘Honeyjar’, with ‘Li’ as the dominant one in the U.S. [22,23].

After more than 180 years of jujube importation into the U.S., there were scattered jujube trees in people’s backyards with varied fruit quality and without proper identity [9]. Jujube trees are common in front yards or backyards in the Fabens/Tornillo area near El Paso, TX (personal communication with Jamie Iglasias). Local people refer to them as aceituna (olive fruit) or asisifus (deformation of *Ziziphus*). Sour jujube trees with tiny round fruits can be found on the New Mexico State University (NMSU) campus, and there is a semi-naturalized jujube population at the NMSU Tucumcari Center. Most jujube cultivars in the U.S. are causally named without detailed information except the directly imported cultivars [9]. Cultivar trials were limited until the recent replicated cultivar trials at multiple locations in New Mexico [9,24,25,26]. The imported cultivars may be renamed by using different methods. Synonyms are common for jujube cultivars, causing confusion among growers when selecting cultivars. Genetic analyses are necessary to accurately identify jujube accessions and clarify the relationship between germplasm and cultivars.

DNA fingerprinting has evolved into an invaluable tool for managing genetic resources and crop genetic improvement [27,28]. Various types of molecular markers have been employed for the management of jujube germplasm, such as amplified fragment length polymorphism (AFLP), simple sequence repeats (SSRs), and single nucleotide polymorphisms (SNPs) [13,15,29,30,31,32]. Genetic diversity and phylogenetic relationship of 255 cultivars of jujube from China were studied by Liu et al. [33] using SSR analysis. Xu et al. [15] genotyped 962 jujube accessions from the two largest Chinese jujube collections: the National Chinese Jujube Germplasm Repository and the National Foundation for Improved Cultivar of Chinese Jujube using 24 SSR markers. Song et al. [30] developed a large panel of single nucleotide polymorphism (SNP) markers and validated 288 SNPs by genotyping 114 accessions of Chinese jujube germplasm and found significant mislabeling.

The most abundant class of polymorphisms in plant genomes is single nucleotide polymorphisms (SNPs). Unlike SSR markers, SNP-based genotyping can be carried out without the need for DNA size separation, allowing for automated high-throughput setups [33]. The diallelic feature of SNPs offers advantages over other methods such as a lower rate of allele calling errors, inter-laboratory compatibility, and cost of effective genotyping. Due to these benefits, SNPs are increasingly preferred as markers for genotype identification and genetic diversity studies in perennial horticultural crops [29,33,34,35]. The objectives of the present study were to assess the genetic integrity and diversity of the jujube germplasm from New Mexico and the southwestern corner of Texas using SNP markers. This is the first study in the U.S. on genotyping jujube germplasm.

## 2. Results

A total of 204 accessions of Chinese jujube were sent for genotyping, and only 186 accessions with <10% missing values were selected for further analysis.

### 2.1. Cultivar/Germplasm Identification

**Figure 1:**
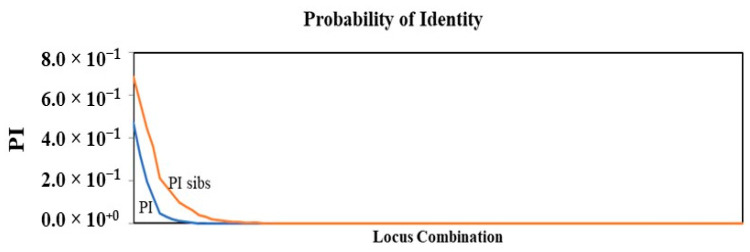
Sibling probabilities of identity (PID-sib) based on 94 SNP markers and 186 jujube accessions from New Mexico and southwestern Texas. The chance that two sibling individuals randomly selected from this collection have identical multilocus genotypes became near to zero after 94 SNP loci were used.

Multiple replications of the SNP profiles from the same jujube cultivars demonstrated remarkable consistency in the genotyping results (Table S1). Multilocus SNP data of all the accessions were shown in Table S1. Accessions that were completely matched at the genotyped SNP loci were considered to share the same genotype or be clones. Pairwise multilocus matching analysis revealed synonymous and anonymous mislabeling among the jujube genotypes. The probability of identity (PID) is an important statistical tool for calculating the approximate number of marker loci required to correctly identify each unique individual [30]. PID-sib, calculated from the 186 samples used in this study, estimated the probability of two unrelated samples having the same genotype at all 94 SNP loci to be 5.8 × 10^−15^ (Figure 1). This indicates that, based on the 94 SNPs, the likelihood of two distinct jujube genotypes in the population having the same genotype was nearly zero. Thus, there is sufficient statistical power for accurate authentication of jujube.

Multilocus matching of the SNP profile revealed a significant rate of duplication among these jujube samples; 48 (or approximately 26%) of the 186 examined accessions can be categorized into 14 synonymous groups (Table 1). Each synonymous group contained two to eight cultivars/accessions. The synonymous group 1 had cultivar ‘Texas Sawmill’, which is synonymous to the popular cultivar ‘Sherwood’, and thus, seven accessions from the Texas samples were cultivar ‘Sherwood’. Only one genotype was selected from each synonymous group and included in the diversity analysis, resulting in 152 genotypes with distinctive SNP profiles.

The descriptive statistics for the 94 polymorphic SNPs across the 152 jujube accessions were computed (Table 2 and Table S2). The mean information index was 0.516, with values ranging from 0.014 to 0.693. The observed heterozygosity ranged from 0.016 to 0.737, with an average of 0.3, whereas the mean expected heterozygosity was 0.34, with a range of 0.012 to 0.500.

### 2.2. Population Structure and Analysis of Molecular Variance (AMOVA)

**Figure 2:**
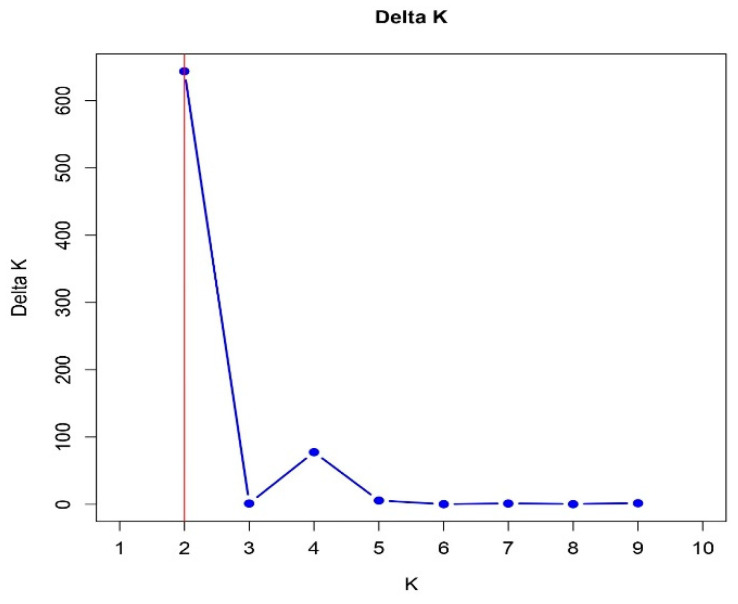
Plot of ΔK (filled circles, blue line) calculated as the mean of the second-order rate of change in likelihood of K divided by the standard deviation of the likelihood of K, m(|L″(K)|/s[L(K)]. Red line shows the most rational value of K based on the Evanno method.

**Figure 3:**
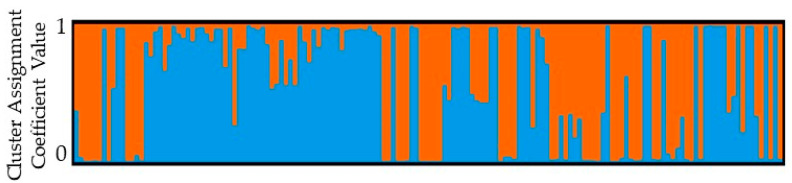
Inferred clusters in the 152 Chinese jujube accessions using Structure Selector. Each vertical line represents an individual multilocus genotype. Each color represents the cluster from which the genotype or partial genotype was produced, suggesting its most likely ancestry. Blue and orange colors represent first and second cluster respectively. Individuals with multiple colors indicate admixed genotypes with contributions from different clusters.

The delta K value, based on Structure Selector [34], indicated that the 152 jujube accessions could be partitioned into two clusters (Figure 2 and Figure 3 and Table S3). With a high assignment coefficient value (Q > 0.80), the first group consisted of 73 core members, while the second group had 49 members. The remaining 30 cultivars were classified as admixed genotypes (Table S4). The first cluster mostly consisted of cultivars from Kentucky (KY), Missouri (MO), and New Mexico. It also included 15 accessions from Texas, 3 from Las Cruces, and 9 from Tucumcari, NM, which had no identity. The second group included jujube accessions from Las Cruces and Tucumcari, NM, and Tornillo and Fabens, TX, but this group did not include any cultivars. All the accessions in this group were sour jujube germplasm (Table S4). The admixture group comprised accessions from Tornillo and Fabens, TX, and Las Cruces and Tucumcari, NM, as well as cultivars from New Mexico, Kentucky, and Missouri states.

**Figure 4:**
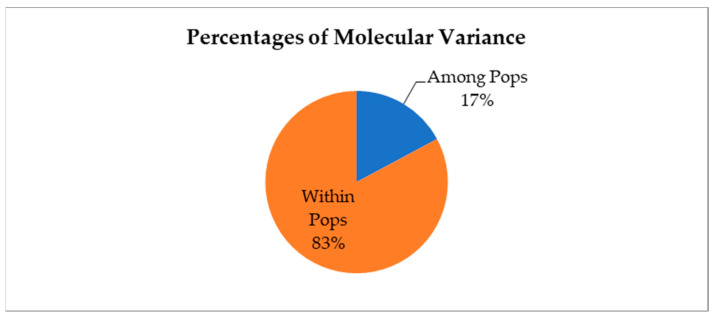
Analysis of molecular variance (AMOVA) of core members in the two jujube groups assigned by the Structure program.

Analysis of molecular variance (AMOVA) conducted with the two groups showed that within-group and among-group variances were both highly significant, accounting for 83% and 17% of total molecular variance, respectively (Figure 4). The pairwise Fst between the two groups was 0.172, with a significance of *p*-value < 0.001 in the permutation test, indicating significant genetic differentiation between the two groups.

### 2.3. Principle Coordinate Analysis and Clustering Analysis

**Figure 5:**
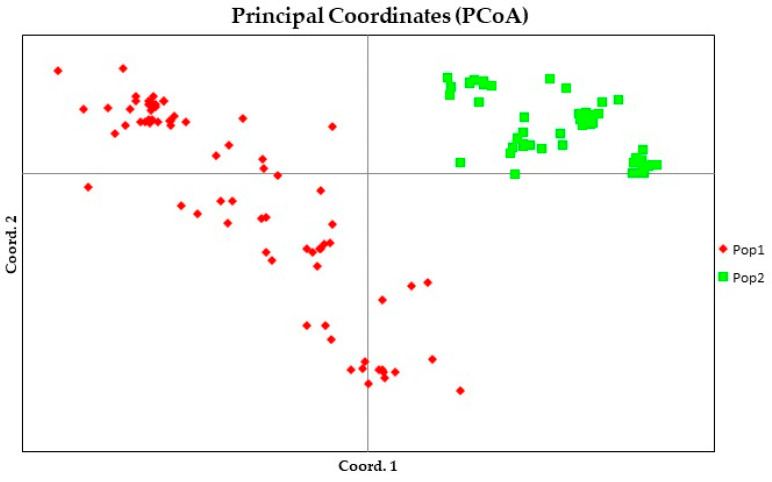
Principle coordinate analysis of the two distinctive jujube groups in 122 accessions. Points in the PCoA plot represent jujube samples, each colored and shaped according to genotype. Red and green points, respectively, represent populations 1 and 2 that comprise cultivar-type jujube and sour-type jujube, respectively.

The principal coordinates analysis (PCoA) demonstrated the genetic relationships between the analyzed jujube accessions (Figure 5) based on the genetic distance matrix. The two core groups assigned by the Bayesian clustering analysis were clearly separated, indicating that these two groups had a distinct genetic background. The percentage of variation explained by the first three axes were 18.84%, 13.05%, and 9.43%, respectively. A total of 41.32% of genetic variation was explained by the top three axes.

**Figure 6:**
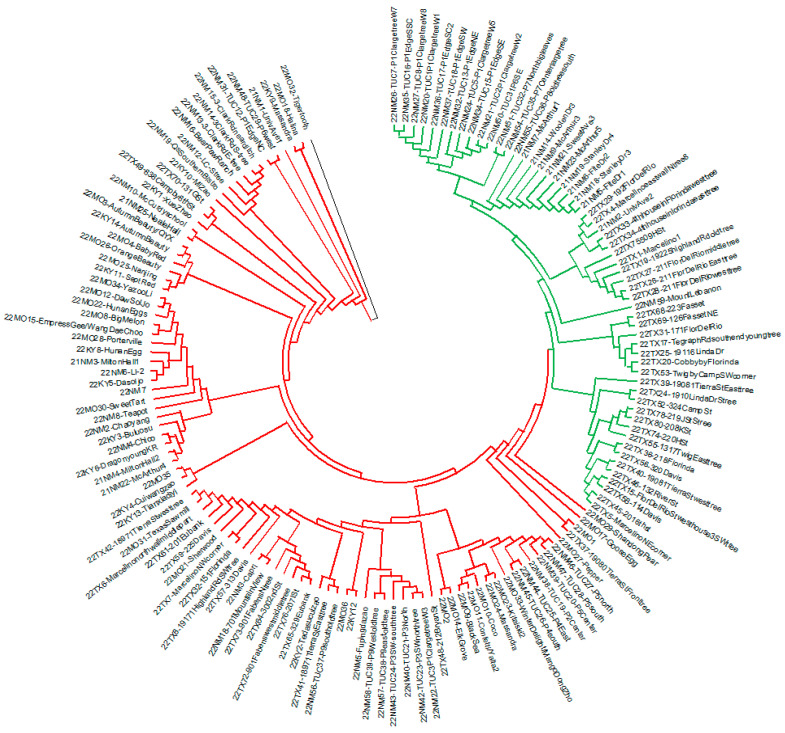
A phylogenetic tree constructed in MEGA 11 software using the neighbor-joining method with 1000 bootstrap replicates of the nucleotide sequences of 152 Chinese jujube accessions from Texas, New Mexico, Missouri, and Kentucky, United States, based on 94 SNP markers. The accessions in red and green colors represent cultivar and sour jujube respectively.

The neighbor-joining phylogenetic tree provided additional complementary information to the findings of PCoA and Bayesian stratification (Figure 6). The NJ tree classified 152 jujube accessions into two main clusters, with multiple subclusters. Each subcluster contained 1–17 samples that were closely related. The first main cluster (shown in red color in Figure 6) aligned with the first group inferred by the structure analysis. It predominantly comprised commercial cultivars from Kentucky, New Mexico, and Missouri with a few accessions from Las Cruces, Gila, and Tucumcari, New Mexico, and Tornillo/Fabens, TX.

Two of the popular cultivar groups in the first cluster are ‘Li’ and ‘Sherwood’. The ‘Sherwood’ group included closely related cultivars ‘Sherwood’, ‘Capri’, and ‘Texas Sawmill’. Most of the large-fruited trees from Fabens/Tornillo, Texas, were classified as ‘Sherwood’ type. ‘Sherwood’ was one of the largest groups in the first cluster, comprising 17 accessions. The other dominant group, ‘Li’, was comprised of cultivars ‘Yazoo Li’, ‘DaeSolJo’, ‘Hunan Eggs’, ‘Big Melon’, ‘Empress Gee/Wang Dae Choo’, and ‘Li-2’. There was also an unidentified germplasm from Las Cruces, New Mexico, that fell into the ‘Li’ group. Six samples from Tucumcari in the cultivar cluster were closely related to ‘Fupingdazao’. Similarly, cultivar pairs such as ‘Black Sea’ and ‘Confetti/Yalta’, as well as ‘Massandra’ and ‘Kitaiski2’, were related. The second cluster (shown in green color in Figure 6) mostly consisted of jujube groups with tiny, spherical fruits from Tornillo and Fabens, Texas, and Tucumcari and Las Cruces, New Mexico, likely representing the sour jujube (*Z. spinosa*). Within this group, nine samples from Las Cruces formed a subcluster. There were also two subclusters from Tucumcari, and eleven subclusters from Texas in the sour jujube group.

Both the cultivar cluster and the sour jujube cluster had some samples classified as admixture according to Bayesian analysis. The results of the phylogenetic tree analysis were consistent with those of the structure analysis.

### 2.4. Fruit Characteristics of Germplasm at Different Locations

#### 2.4.1. Fabens/Tornillo, TX

**Figure 7:**
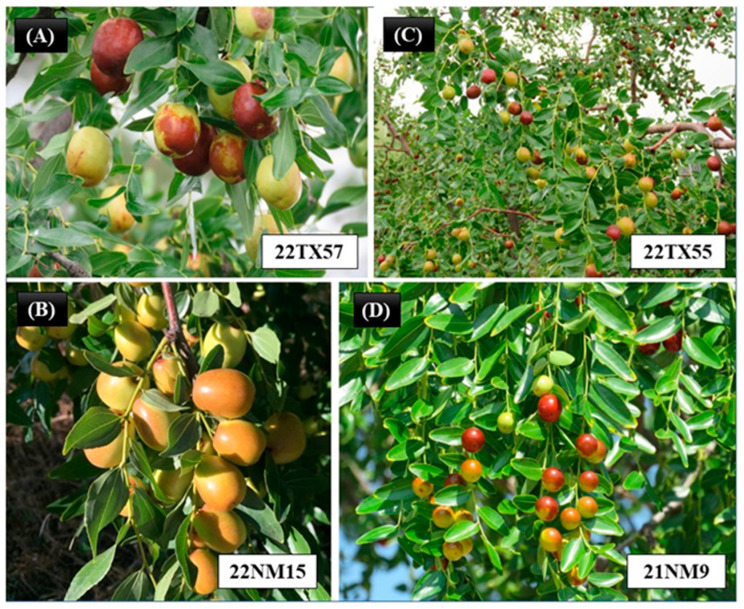
Pictures of fruits from different locations: (**A**) Sherwood-type jujube fruit of southwestern Texas, (**B**) cultivar-type jujube fruit of the Gila/Silver City area, (**C**) sour-jujube-type fruit in southwestern Texas, and (**D**) sour jujube fruit near the NMSU family housing area, Las Cruces, NM.

There were two types of jujube fruits in the Fabens and Tornillo area of Texas: (1) sour jujube types with small round fruit and long decorative branchlets or medium elongated fruit and (2) large-fruited accessions that were closely related to the commercial cultivar ‘Sherwood’ (Figure 7).

#### 2.4.2. Las Cruces, NM

The jujube trees at NMSU campus were dominated with the sour jujube type near the family housing area, and a few cultivar trees were scattered on campus. The fruits of sour jujube type were tiny, spherical in shape, and sour in taste.

#### 2.4.3. Gila/Silver City, NM

Almost all the accessions from this area exhibited similar fruit morphology characterized by medium fruits and heavy bearings (Figure 7). The original two trees at LC Ranch headquarters in Gila might have spread through sucker transplanting to the Gila/Cliff/Silver City area.

## 3. Discussion

Despite significant advances in plant genomics research, modern molecular tools for jujube germplasm management are not widely utilized. Cultivar mislabeling and anonymous and synonymous accessions are the major challenges for jujube researchers and growers. Currently in the United States, there is a lack of research conducted to assess the genetic integrity of jujube cultivars/selections. In the present study, we genotyped a panel of cultivated and sour jujube germplasm using 94 SNP markers to identify the accessions, evaluate the genetic diversity, assess population structure, and identify labeling errors.

SNP genotyping has an edge over other DNA fingerprinting techniques as it allows for accurate multilocus genotype matching at a lower cost. It provides the most direct scientific evidence for cultivar authenticity. Additionally, this technology enables rapid processing and analysis of large number of samples through high-throughput genotyping, while delivering highly reliable and consistent results.

### 3.1. Cultivar Identification

Accurate jujube cultivar identification is essential for effective management of jujube genetic resources, propagation of planting materials, and selection and breeding for cultivars with favorable agronomic traits and qualitative characteristics.

The number of genetic loci for genotyping determines the accuracy and effectiveness of genetic identification through SNP fingerprints. Probability of identity (PID) is an important statistical measure for estimating the number of loci necessary to accurately identify all unique individuals [30]. Accumulated PID values can be computed by multiplying single-locus PID values together, considering loci independence. Because domesticated crop species such as jujube may have closely related parents, a PID determined for siblings would offer a stringent criterion for a crop species that has a long history of domestication. The accumulated PID is 5.8 × 10^−15^, showing the strong statistical power of applying this low-density SNP array for jujube genotype identification.

Several studies have documented various rates of duplication in melon [35], grape [36], lychee [37], tea [38], and soybean [39]. The current findings also demonstrated a significant level of genetic redundancy in this jujube collection. This result is similar to the findings of Xu et al. [15] and Song et al. [30], who found that 47% and 43% of the studied Chinese jujube germplasm accessions, respectively, contained at least one duplicated accession. This high prevalence of synonymous mislabeling can be attributed to cultivar renaming following imports from different jujube-growing countries. Some of the duplicates discovered were well-known synonymous cultivars. For example, ‘Sherwood’ is a popular cultivar here in the United States that is synonymous to the cultivar ‘Texas Sawmill’. The same cultivar might have been labeled differently in different places.

It ought to be pointed out that identical SNP profiles, based on 94 SNP markers, may not necessarily guarantee the genetically identical genotype. Somatic mutations are not rare events in jujube, and these mutations may affect plant phenotypic characteristics such as fruit skin color, flesh color, growth behavior, and fruit quality traits [30]. Therefore, caution needs to be taken while making an interpretation. The genotyping projects of apple [40], pineapple [41,42], and banana [43] also faced this issue. More thorough genomic approaches might be used to identify which genes or allele modifications are responsible for the phenotypic variability. It is still crucial to compare phenotypic characteristics among members of the same synonymous groups to complement the results of DNA fingerprinting.

### 3.2. Genetic Relationship among Germplasm Groups

Bayesian stratification divided our germplasm into two populations (Figure 3), i.e., the cultivar jujube population and the sour jujube population. In addition, the AMOVA Fst value of 0.172 demonstrated a clear distinction of these two populations (Figure 4).

The admixture group consisted of cultivars such as ‘Pepper’, ‘Teapot’, and ‘Sweet tart’. It also had accessions from Tucumcari and Texas. As most jujube cultivars were selections from existing germplasm, their origins were mostly unknown, making it difficult to trace their lineage. However, for the admixture accessions from Fabens/Tornillo, TX, it is possible that they resulted from open pollinations between *Z. spinosa* and *Z. jujuba*, as both species coexisted in that area. Figure S1 shows the pictures of *Z. spinosa* and admixture accessions of 22TX53 and 22TX69. The fruit of *Z. spinosa* typically has thin flesh, and it has smooth hard skin with no wrinkles. The thin flesh layer remains attached to the skin when the fruit is dried, and there is a gap between the seed and the fruit skin in dry fruit. In contrast, the fruit of admixture accessions are slightly larger, have a brighter color, and exhibit wrinkles, indicating different flesh texture compared to *Z. spinosa*. There was no gap between the seed and the skin in dry fruit. The fruit of admixture accessions tasted better than *Z. spinosa,* which explains why they were retained in that area. However, due to human selections in Fabens/Tornillo, there are few original bush-type jujubes with small, round fruit and highly thorny sour-jujube-type accessions. Instead, there are now tree types with spherical, tasty fruit. The Tucumcari accessions would be similar, but we have less information about them, as those trees lacked basic maintenance and were in survival mode. They could originate from open pollinations between sour jujube and cultivar types in Chico, CA, before they were planted in Tucumcari, NM; alternatively, it was also possible they originated from seeds of open-pollinated fruit between the two species at Tucumcari, NM.

The Evanno Delta K graph (Figure 2) also indicated a secondary peak at K = 4. The related partitioning outcomes are, therefore, presented in Figure S2. At K = 4, however, a much larger proportion of the cultivars were classified as admixture. The results at K = 4 demonstrated that our samples were divided into four populations. However, the four subpopulations were the result of partitioning of the two subpopulations. At K = 4, both the sour jujube and cultivar populations were further split into two subpopulations each. The ‘Li’ and ‘Sherwood’ types were the further clusters of the cultivar-type subpopulation. A larger sample size could have justified the partitioning results at K = 4.

The NJ tree provided complementary information about the relationship among the jujube accessions (Figure 6). Two big clusters and multiple subclusters were denoted by the NJ tree. The two big clusters resembled the two subpopulations inferred from the structure analysis.

### 3.3. A Unique Jujube Population at Fabens/Tornillo Area, TX

According to historical accounts, a Chinese railroad workers’ camp existed in the late 19th century close to the Fabens/Tornillo area of Texas (personal communication with Jamie Iglasias). Jujube fruit was a part of the diet for those Chinese workers. The littered seeds germinated and grew into trees. It was possible they also brought in sour jujube seeds/fruit. Following 130–140 years of selection, those with large fruit and good fruit flavor were retained and disseminated among family and friends. Most of the trees were planted nearby in Fabens and Tornillo, Texas. It became less popular in towns north of Tornillo. It was probable that jujube trees here were transported to other locations or states.

Old jujube trees at 108 Capri, Las Cruces, 701 Mountain View, and Silver City had identical SNP sequences to those Sherwood-like trees. In Fabens/Tornillo, there were more Sherwood-related accessions, albeit not identical to Sherwood germplasm. It is difficult to determine whether Sherwood or the Sherwood-like germplasm existed earlier.

The *Z. spinosa*-type accessions at Fabens/Tornillo were distinct, characterized by long decorative branchlets and small, yet tasty, fruit, which likely contributed to their preservation. Multiple subclusters of *Z. spinosa* type in the Fabens/Tornillo were observed. However, we did not identify any popular commercial cultivars like ‘Li’ in this area. We only focused on the jujube germplasm specifically in Fabens/Tornillo, TX, and it is possible there are other jujube populations in other states/locations near old Chinese railroad or mine workers’ camps that await exploration.

So, we assumed the jujube population of large-fruited Sherwood-like accessions and *Z. spinosa* types is a unique jujube population, native to the Fabens/Tornillo area, Texas. Evaluating those large-fruit accessions and sour jujube types and recommending high-performing accessions to growers would be interesting research topics.

### 3.4. Semi-Naturalized Jujube Population at Tucumcari, NM

Our results indicate there were both *Z. spinosa* types and *Z. jujub*a types for that semi-naturalized jujube population in Tucumcari, NM. The sour jujube types could be rootstocks that spread out by themselves, while the cultivars could be the original planted trees; they self-rooted and spread out. It is also possible that just the rootstocks survived after the original trees died. This population, we presume, was from Frank Meyer’s importation at the Chico Plant Introduction Station [44]. The trees at Tucumcari, in contrast to those accessions from Fabens/Tornillo, were on dry land without irrigation and with very limited growth each year. It would be harder to get scion wood and evaluate those accessions.

## 4. Materials and Methods

### 4.1. Jujube Germplasm Sampling

**Figure 8:**
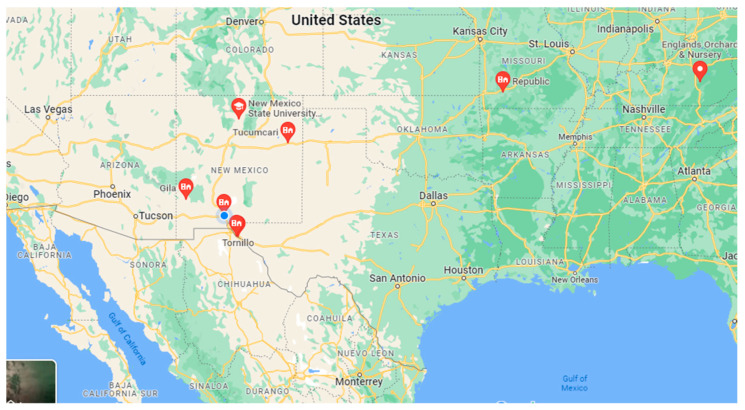
Jujube germplasm collection sites.

Jujube leaf samples were collected from Tornillo, TX: 31°26′22″ N, 106°5′44″ W, Fabens, TX: 31°30′18″ N, 106°9′15″ W, NMSU, Las Cruces, NM: 32°16′58.8″ N, 106°44′52.8″ W, Gila, NM: 32°57′58″ N, 108°34′37″ W, Silver City, NM: 32°46′41″ N, 108°16′27″ W, and Tucumcari, NM: 35°10′10″ N, 103°43′32″ W. The commercial cultivars were obtained from Republic, MO: 37°7′18″ N, 93°28′17″ W, England’s Orchard and Nursery, McKee, KY: 37°25′9″ N, 83°59′37″ W, and Alcalde, NM: 36°5′17″ N, 106°3′25″ W in the United States (Figure 8). For genotyping analysis, 6–12 fresh healthy leaves were sampled and inserted into a small bag with silica desiccant. After drying, samples were kept in the freezer.

New Mexico State University campus at Las Cruces, NM: there are sour jujube trees (*Z. spinosa* Hu.) near the university family housing and several large-fruited jujube trees of *Z. jujuba* Mill. near several buildings at NMSU. A total of 25 trees were sampled in the fall of 2021.

Tucumcari, New Mexico: there were eight naturally spread jujube patches that emerged after the original USDA jujube trials were abandoned in the 1930s (personal communication with Leonard Lauriault). These patches varied in size from 30–50 trees to half an acre in size at the New Mexico State University Tucumcari Science Center, Tucumcari, NM. Those trees were on dry land without irrigation, and they spread naturally and survived for nearly 90 years. In some large patches, the original trees could be identified in the central part of the patch. Due to the huge number of trees, not every tree was sampled. The original trees and trees from the edges in different directions for large patches and fewer trees from small patches were sampled, resulting in a total sample number of 39 from 8 patches.

Gila/Silver City, NM: there are two jujube trees that were planted around 1900 at the headquarters of LC Ranch (personal communication with Gretchen VanAuken). There are scattered jujube trees near the Gila/Cliff area and Silver City, NM, with their presence even stretching to Mimbres, NM. Nine samples were collected from the Gila/Silver City area in June 2022.

Alcalde/Espanola, NM: nine jujube cultivar leaf samples were collected at Alcalde, NM, and one sample from a jujube tree at McCurdy School in Espanola, NM, in June 2022.

Fabens/Tornillo near El Paso, TX: jujube trees are commonly found in front yards or backyards of residents in the Fabens/Tornillo area. We drove from street to street and sampled all accessible jujube trees with a total of 79 samples in June 2022, which should be representative of the jujube population in the Fabens/Tornillo area.

We also collected jujube cultivars from jujube enthusiasts Michael Nave in Republic, MO, and Cliff England at McKee, KY, for a total of 43 samples.

A total of 204 jujube accessions were sent to the Sustainable Perennial Crops Laboratory, USDA-ARS, Beltsville Agricultural Research Center, for genotyping.

### 4.2. DNA Extraction

DNA was extracted from the dried jujube leaves using the DNeasy Plant Mini Kit (Qiagen Inc., Valencia, CA, USA), which relies on using silica as an affinity matrix. As described by Fang et al. [38] and Song et al. [30], a TissueLyser II (Qiagen Inc.) was used to disrupt the dry leaf tissue samples with high-speed shaking (30 Hz for 1 min) using a Lysing Matrix A (MP Biomedicals, Solon, OH, USA). Thermo Scientific’s (Wilmington, DE, USA) NanoDrop spectrophotometer was used to measure DNA concentration by absorbance at 260 nm and to evaluate DNA purity at ratios of 260:280 and 260:230.

### 4.3. SNP Markers and Genotyping

The Assay Design Group at Fluidigm Corp. (South San Francisco, CA, USA) used the 94 markers, for which the putative SNP primers were designed from the 192 polymorphic SNPs that were selected based on their no-call rate and genotyping consistency, enabling bi-allelic scoring of SNPs at specific loci (KBioscience Ltd., Hoddesdon, UK) by Song et al. [30]. The core set of 94 markers were selected based on information index, linkage disequilibrium (LD) values, and wide distribution of those markers throughout the 12 chromosomes (Table S5). For genotyping, the Fluidigm SNP type Genotyping Reagent Kit and a nanofluidic 96.96 Dynamic Array integrated fluidic circuit were employed. The genotyping process was conducted using the high-throughput Fluidigm EP1 system following the manufacturer’s specifications (IFC, Fluidigm Corp.). This system automates PCR reaction assembly, enabling simultaneous testing of 96 samples with 96 SNP markers. Fluorescent images of the endpoint reactions in the 96.96 IFC were captured using an EP1 imager (Fluidigm Corp., South San Francisco, CA, USA) and analyzed using Fluidigm Genotyping Analysis Software (Fluidigm Corp., South San Francisco, CA, USA).

### 4.4. Data Analyses

Pairwise multilocus matching across all the individual samples was employed to identify duplicate accessions. DNA samples that were perfectly matched at all genotyped SNP loci were designated the same cultivar or clones. The program GenAlEx 6.5’s multilocus matching algorithm was used to perform the computation [45].

Redundant samples were eliminated, retaining only one genotype from each duplicate group. The nonredundant samples were then used for the subsequent diversity study. Summary statistics of the SNP markers such as minor allele frequency, observed heterozygosity, expected heterozygosity, and Shannon’s information index were calculated using the GenAlEx 6.5 program [45]. The probability of the identity of siblings (PID-sib) [46] was calculated to determine the genotyping potential of the jujube SNP panel. The PID-sib refers to the probability that two randomly selected from a population will share the same multilocus genotype. The overall PID-sib delivers the minimum number of loci needed to identify all individuals, including relatives, and represents the upper limit of the PID ranges that can exist in a population.

The SNP data were further analyzed using the model-based Bayesian clustering algorithm using Structure v2.3.4 software to determine the population structure [47]. This algorithm aimed to identify genetically different subpopulations based on allele frequencies. An admixture model was applied. The number of genetic clusters (K value) was set from 1 to 10. For each given number of clusters (K value), 10 separate runs, each with 100,000 iterations following a burn-in of 200,000, were performed. All the accessions were considered to have unknown origins. The Delta K value was used to determine the most rational number of clusters using the online Structure Selector program [34,48,49].

The individual data were subjected to a distance-based multivariate analysis. Using the distance option in the GenAlEx 6.5 program, pairwise genetic distances were calculated. Principle coordinate analysis (PCoA) was also computed using the same computer software [50]. To further illustrate the genetic relationships among the cultivars with distinct SNP profiles, cluster analysis based on the neighbor-joining method was employed. A phylogenetic tree was generated using Mega 11 software with 1000 bootstrap replicates [51].

## 5. Conclusions

In conclusion, we genotyped jujube germplasm from multiple locations in NM and the southwestern corner of TX using 94 SNP markers through a nanofluidic genotyping system. We also identified the genetic relationship between existing jujube cultivars in the United States. This is the first research to employ molecular makers to authenticate jujube germplasm in the United States. The availability of efficient cultivar identification technologies is crucial for growers and researchers in selecting and identifying cultivars accurately. Furthermore, it is vital to understand the genetic relationships among jujube accessions for their effective utilization as breeding materials. We are currently conducting additional research to enhance our findings, particularly through study of morphological characteristics.

## Figures and Tables

**Table 1:** List of 48 jujube accessions of 14 synonymous groups identified by SNP markers. Accessions in bold were retained for diversity analysis.

Synonymous Group	Accession	Synonymous Group	Accession
1	22TX9-19171 Highland Rd-SE tree	8	**21NM6-313 Fite Dr.**
1	22TX3-Marcelino Nursery-sample 3	8	21NM20-1541 Standley Dr.
1	22TX2-Marcelino Nursery-sample 2	8	21NM11-521 Sweet Ave
1	22TX18-19280 Linda Dr.		
1	22TX16-Flor Del Rio St, west house3-north tree	9	22TX66-2nd by Fasset SE 2nd lot-west tree
1	22TX14-540 Hemley St west neighbor-south tree	9	22TX67-2nd by Fasset SE 2nd lot-middle tree
1	22TX13-540 Hemley St west neighbor-north tree	9	**22TX68-223 Fasset**
1	**22MO31 (Texas Sawmill)**		
		10	22TX11-16772 Drake b 4th St-north tree 2
2	**22TX61-201 Eubank**	10	22TX22-19092 Cobby St
2	22TX60-211 Eubank	10	22TX30-190 Flor Del Rio
2	22TX44-115 6th Street-north tree	10	22TX38-19080 Tierra St-back tree
2	22TX21-opposite side of 19125 Cobb St	10	22TX50-Resinger Park Trailer-east tree
		10	22TX51-Resinger Park Trailer-west tree
3	**22TX59-225 Davis**	10	22TX54-1317 Twig-west tree
3	22TX10-19171 Highland Rd-NE tree	10	**22TX58-114 Davis**
4	**22NM35-TUC16 Patch 1 Edge, SSC**	11	**22TX52-324 Camp Street**
4	22NM30-TUC11 Patch 1 Edge, NW	11	22TX35-opposite side of 211 Florinda St
4	22NM29-TUC10 Patch 1 Edge, west		
4	22NM28-TUC9 Patch 1 C, west 9	12	22TX12-540 Hemley St
4	22NM23-TUC4 Patch 1 C, west 4	12	**22TX20-Cobby by Florinda, opposite side of 19171**
5	**21NM5-Fite Dr. House #309**	13	22TX23-1910 Linda Dr-north tree
5	21NM13-1010 Wooten Dr.	13	**22TX31-171 Flor Del Rio**
6	22TX77-1101 Fabens west tree	14	22NM52-TUC33 Patch 7, west
6	**22TX78-219 J St-south tree**	14	22NM53-TUC34 Patch 7, east
		14	**22NM54-TUC35 Patch 7, Center**
7	**21NM19-1536 Standley Dr.**		
7	21NM17-1500 Standley Dr.		

**Table 2:** Shannon’s information index, heterozygosity, and inbreeding coefficient of the 94 SNP loci, scored on 152 jujube accessions.

Category	Shannon’s Information Index	Observed Heterozygosity	Expected Heterozygosity	Inbreeding Coefficient
Range	0.014–0.693	0.016–0.737	0.012–0.500	−0.476–0.811
Mean	0.516	0.300	0.340	0.118
SE	0.014	0.016	0.012	0.033

## Data Availability

Data will be available upon request.

## Supplementary Materials

The following supporting information can be downloaded at https://www.mdpi.com/article/10.3390/plants12132405/s1: Table S1: SNP based DNA fingerprints generated by the 94 SNP markers for 186 Chinese jujube accessions; Table S2: Minor allele frequency, information index, heterozygosity, and inbreeding coefficient of the 94 SNP loci scored on 152 Chinese jujube accessions; Table S3: Delta K at different values of K shown by structure program; Table S4: Bayesian Stratification; Table S5: Detailed information of the 94 markers used for genotyping jujube germplasm; Figure S1: *Z. spinosa* fruit vs. accessions of admixture fruit with brighter fruit color and more wrinkles (thick flesh) on fruit surface. From left to right: *Z. spinosa* fruit from NMSU campus 21NM9, admixture accessions of 22TX53 and 22TX69; Figure S2: Structure partitioning at K = 4.
