# Supplementary material for: Genotyping of Jujube (Ziziphus spp.) Germplasm in New Mexico and Southwestern Texas
Source: Plants (Basel). 2023 Jun 21;12(13):2405. doi: 10.3390/plants12132405 (PMC10346288; doi:10.3390/plants12132405)
Supplement: Supplementary file 1 [file plants-12-02405-s001.zip › Figure S1.pptx]

## Slide 1
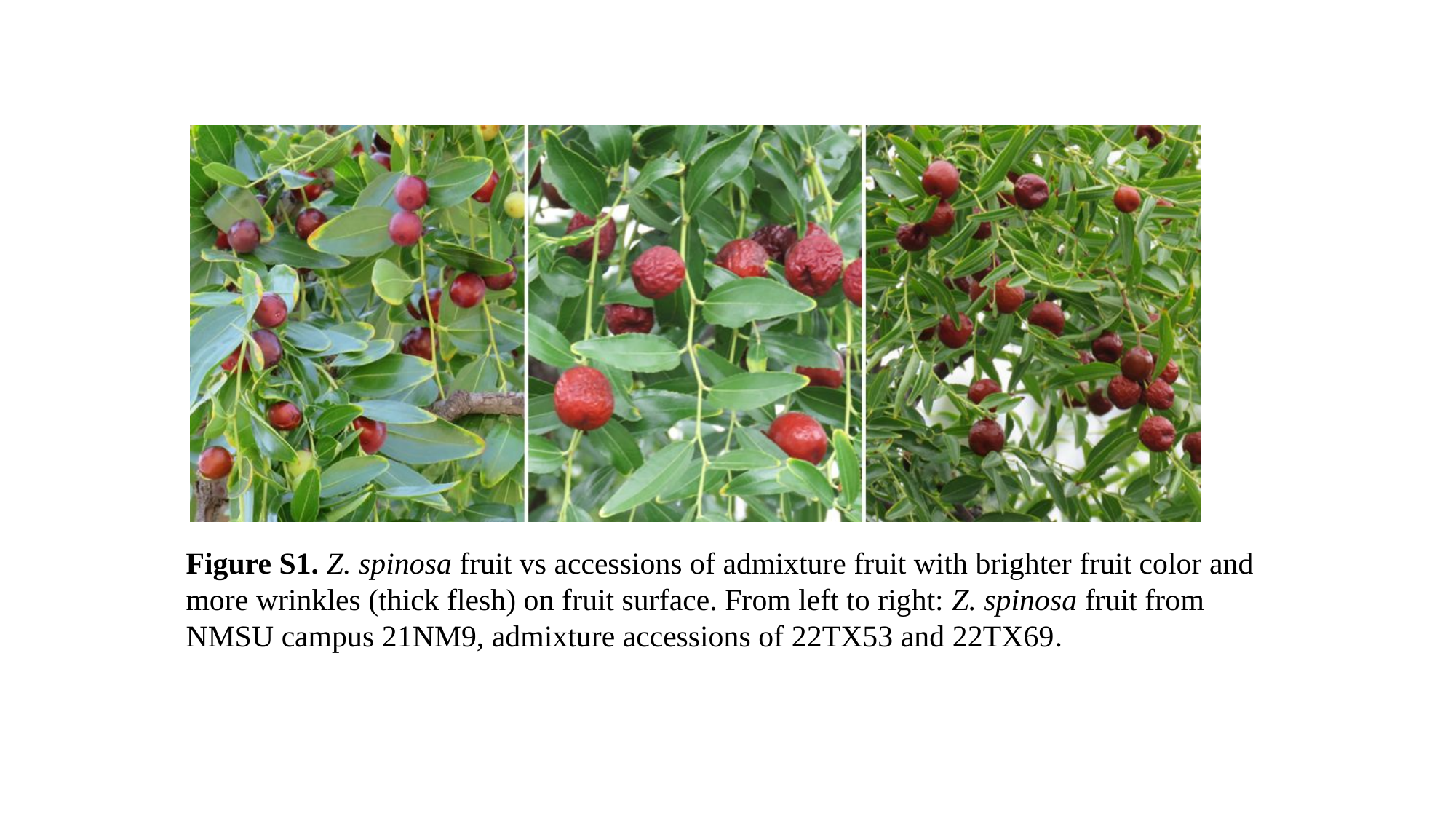

Figure S1. Z. spinosa fruit vs accessions of admixture fruit with brighter fruit color and more wrinkles (thick flesh) on fruit surface. From left to right: Z. spinosa fruit from NMSU campus 21NM9, admixture accessions of 22TX53 and 22TX69.
