# Supplementary material for: Genotyping of Jujube (Ziziphus spp.) Germplasm in New Mexico and Southwestern Texas
Source: Plants (Basel). 2023 Jun 21;12(13):2405. doi: 10.3390/plants12132405 (PMC10346288; doi:10.3390/plants12132405)
Supplement: Supplementary file 1 [file plants-12-02405-s001.zip › Figure S2.pptx]

## Slide 1
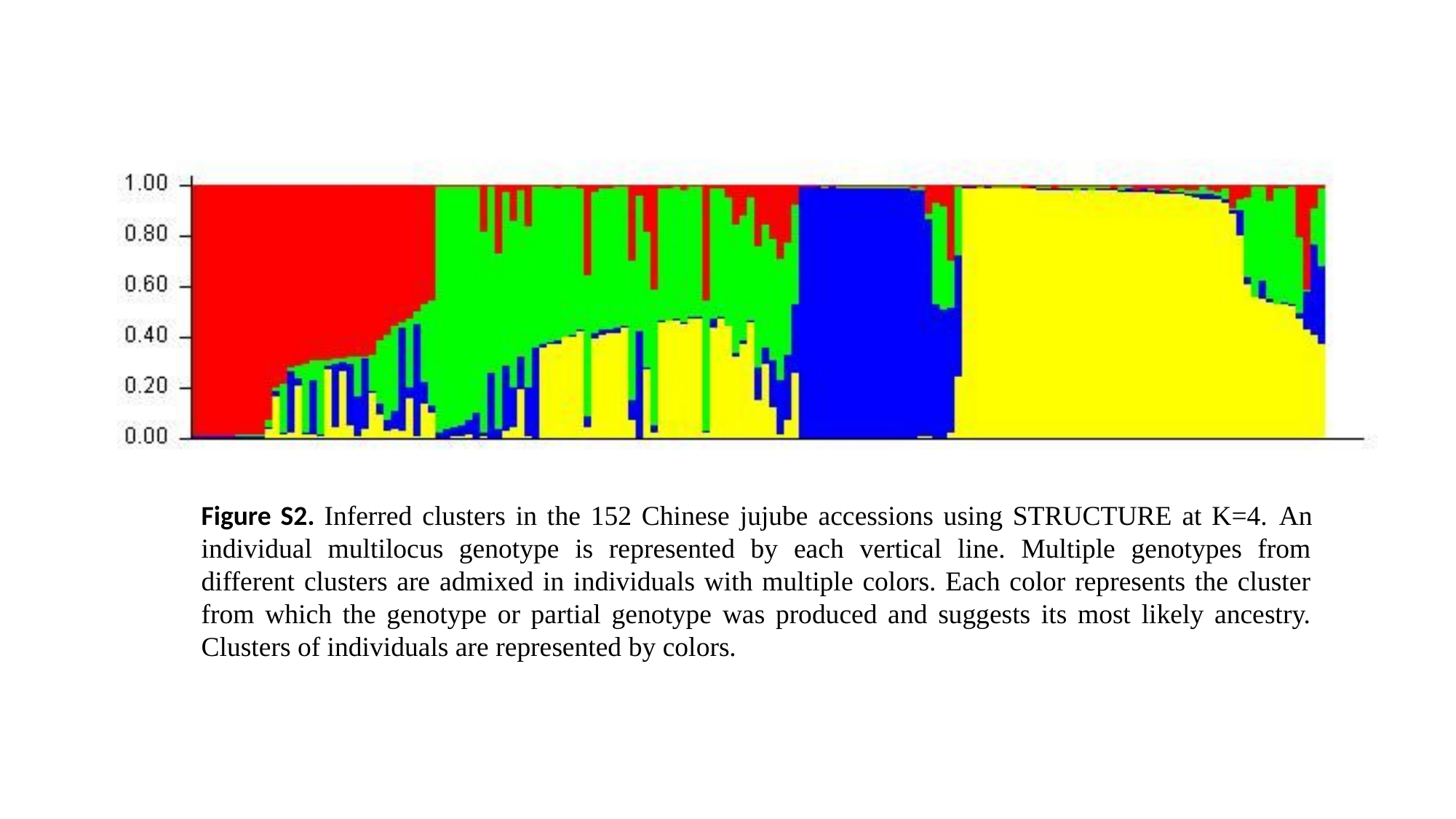

Figure S2. Inferred clusters in the 152 Chinese jujube accessions using STRUCTURE at K=4. An individual multilocus genotype is represented by each vertical line. Multiple genotypes from different clusters are admixed in individuals with multiple colors. Each color represents the cluster from which the genotype or partial genotype was produced and suggests its most likely ancestry. Clusters of individuals are represented by colors.
